# Impaired T3 uptake and action in MCT8-deficient cerebral organoids underlie Allan-Herndon-Dudley syndrome

**DOI:** 10.1172/jci.insight.174645

**Published:** 2024-02-20

**Authors:** Federico Salas-Lucia, Sergio Escamilla, Antonio C. Bianco, Alexandra Dumitrescu, Samuel Refetoff

**Affiliations:** 1 Section of Adult and Pediatric Endocrinology, Diabetes and Metabolism, Department of Medicine, The University of Chicago, Chicago, Illinois, USA.; 2 Instituto de Neurociencias de Alicante, Miguel Hernández-CSIC University, Sant Joan d’Alacant, Alicante, Spain.; 3 Committee on Molecular Metabolism and Nutrition,; 4 Department of Pediatrics, and Committee on Genetics, The University of Chicago, Chicago, Illinois, USA.

**Keywords:** Endocrinology, Neuroscience, Neurodevelopment, Thyroid disease, Transport

## Abstract

Patients with mutations in the thyroid hormone (TH) cell transporter monocarboxylate transporter 8 (*MCT8*) gene develop severe neuropsychomotor retardation known as Allan-Herndon-Dudley syndrome (AHDS). It is assumed that this is caused by a reduction in TH signaling in the developing brain during both intrauterine and postnatal developmental stages, and treatment remains understandably challenging. Given species differences in brain TH transporters and the limitations of studies in mice, we generated cerebral organoids (COs) using human induced pluripotent stem cells (iPSCs) from MCT8-deficient patients. MCT8-deficient COs exhibited (i) altered early neurodevelopment, resulting in smaller neural rosettes with thinner cortical units, (ii) impaired triiodothyronine (T3) transport in developing neural cells, as assessed through deiodinase-3–mediated T3 catabolism, (iii) reduced expression of genes involved in cerebral cortex development, and (iv) reduced T3 inducibility of TH-regulated genes. In contrast, the TH analogs 3,5-diiodothyropropionic acid and 3,3′,5-triiodothyroacetic acid triggered normal responses (induction/repression of T3-responsive genes) in MCT8-deficient COs, constituting proof of concept that lack of T3 transport underlies the pathophysiology of AHDS and demonstrating the clinical potential for TH analogs to be used in treating patients with AHDS. MCT8-deficient COs represent a species-specific relevant preclinical model that can be utilized to screen drugs with potential benefits as personalized therapeutics for patients with AHDS.

## Abstract 2

MCT8-deficient brain organoids exhibit impaired T3 signaling, which can be recovered with the T3-analogs DITPA and TRIAC.

## Introduction

Thyroid hormones (THs) are crucial for brain development and greatly influence brain function throughout life (1–5). TH relies on specific cell membrane transporters to enter the brain and neural cells, including the monocarboxylate transporter 8 (MCT8; encoded by *SLC16A2* in the X chromosome) (6). MCT8 plays a critical role in TH signaling, as showcased by the profound phenotype observed in boys carrying loss-of-function mutations in *SLC16A2*, which is indicative of brain hypothyroidism during critical developmental stages. Patients with Allan-Herndon-Dudley syndrome (AHDS) exhibit characteristic serum TH abnormalities (high triiodothyronine [T3], low thyroxine [T4], and reverse T3, with normal or slightly elevated thyrotropin) accompanied by severe and irreversible neurological deficits (7), presumably due to reduced TH availability to neural cells. This assumption is mainly circumstantial but also derived from a study that identified an approximately 50% reduction in TH contents in the cerebral cortex and abnormalities in neuronal differentiation, synaptogenesis, and myelination in brain sections of a fetus with AHDS (8, 9). There are also MRI studies indicating hypomyelination during the first years of life (8, 9), but it is unclear whether it persists into adulthood (10).

To gain greater insight into the pathophysiology of AHDS, researchers have looked into the brains of animal models expressing a nonfunctional MCT8 and studied neural cells derived from induced pluripotent stem cells (iPSCs) and found that MCT8 plays a role in the passage of TH through the blood-brain barrier (11–13). Hence, the concept that MCT8 mediates TH transport into the brain parenchyma is well accepted. Notwithstanding, the fact that MCT8 is widely expressed in the human brain (13–16) supports a broader role for MCT8 in T3 transport into neural cells. This seems to be the case in mouse neurons, where Mct8 is critical in an endosomal pathway that retrogradely transports T3 from distal axons to the cell nucleus of these cells (17). Another example is the neural progenitor cells of chicken embryos and adult mouse hippocampi, where Mct8 functions as a gatekeeper in the process of neurogenesis (18, 19). However, a unifying hypothesis for the contribution of MCT8 to TH transport and signaling in human neural cells and how its loss-of-function mutations relate to the neurological manifestations seen in patients is missing.

The treatment of patients with MCT8 deficiency remains challenging (20), and daily care poses a heavy burden on caregivers (21). Current options can only ameliorate thyroid test abnormalities associated with hypermetabolism without improvement of the severe neurologic defects. MCT8 deficiency cannot be corrected with high doses of TH because of its insufficient availability to the brain (22, 23). Thus, studying TH analogs for their potential benefit in treating MCT8-deficient patients is paramount. In particular, the TH analogs 3,5-diiodothyropropionic acid (DITPA) and 3,3′,5-triiodothyroacetic acid (TRIAC) do not depend on MCT8 for transport and have thyromimetic activity by binding to the TH receptors (24, 25) and effect in Mct8-deficient mice (26, 27). DITPA also improves circulating T4 and T3 levels in MCT8-deficient humans (28) and promotes differentiation and myelination in healthy human oligodendrocytes (29). However, we still lack vital information about the role played by MCT8 and potential TH analogs in the human brain because of species differences in brain TH transporters.

To avoid these limitations, we generated cerebral organoids (COs) from patient-derived iPSCs and demonstrated that MCT8 is critical for early neurodevelopment, playing a role in the T3 uptake of developing human neural cells. The resulting impairment in transmembrane transport affects intracellular T3 action, including the expression of genes involved in cerebral cortex development and neural cell maturation. The present investigation reveals that DITPA and TRIAC can act in human neural cells, bypassing a nonfunctional MCT8 and triggering responses similar to those induced by T3. These findings expand our understanding of the pathophysiology of AHDS and have broad implications for devising new potential treatments to improve the quality of life of patients with AHDS.

## Results

### Generation of control and MCT8-deficient COs.

**Figure 1:**
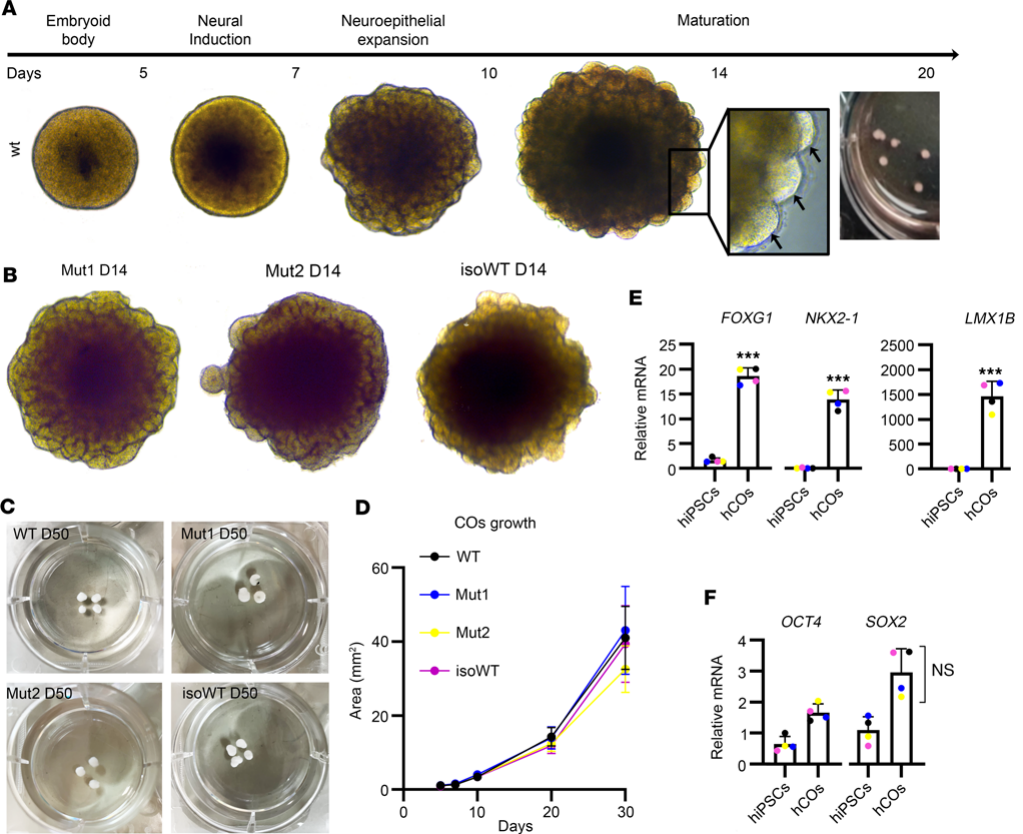
Generation and characterization of control and MCT8-deficient COs. (**A**) Schematic of COs’ generation and timing. COs’ generation started with the culture of EBs, followed by neural induction, neuroepithelial bud expansion, and maturation of the COs. Arrows point to expanded neuroepithelia as evidenced by the EB surface budding. (**B**) Mut1, Mut2, and isoWT COs exhibit neuroepithelial expansion. (**C** and **D**) Control and MCT8-deficient COs formed cerebral like structures (**C**) of similar size (**D**). (**E** and **F**) Relative gene expression (normalized to GAPDH) as determined by quantitative PCR from D20 COs of CNS markers (*FOXG1*, *NKX2.1*, *LMX1B*) (**E**) and of pluripotency (*SOX2*) and ectoderm (*OCT4*) markers (**F**); *n* = 5–6 RNA samples, each of them consisting of 4 pooled COs from either WT or MCT8-deficient COs. Two-tailed Student’s test for comparing human iPSCs versus human COs, and 1-way ANOVA and Tukey test were used for multiple comparisons (growth and SOX2 expression between COs); ***P < 0.001.

We generated COs from 4 human iPSC lines, 2 MCT8-deficient (Mut) and 2 healthy controls (WT): (i) Mut1, from a patient with a nonsense frameshift mutation A404fs416X (amino acids numbered according to the long MCT8 isoform), leading to a premature stop codon; (ii) Mut2, from a patient with the missense mutation P321L; (iii) WT, control cell line from the father of the patient providing Mut2; and (iv) isoWT, control isogenic line in which the mutation of Mut2 has been corrected by CRISPR/Cas9. (Supplemental Figure 1, A and B; supplemental material available online with this article; https://doi.org/10.1172/jci.insight.174645DS1). The 4 cell lines produced uniform round embryoid bodies (EBs) with smooth edges and diameters of about 500 μM and then developed to form optically translucent edges, indicating neural induction (Figure 1A). During the next 10 days in culture, control and MCT8-deficient EBs (Figure 1, A and B) showed neuroepithelial bud expansion and matured until they formed cerebral like structures (Figure 1C), exhibiting similar growth (Figure 1D). At day 20 (D20) of culture, control and MCT8-deficient COs showed high expression of the forebrain markers *FOXG1*, *NKX2-1*, and *LMX1B* at significantly higher levels than the original human iPSCs (Figure 1E) while maintaining a high expression of the pluripotency markers *OCT4* and ectoderm marker *SOX2* (Figure 1F).

### MCT8-deficient CO exhibits impaired early neurogenesis.

**Figure 2:**
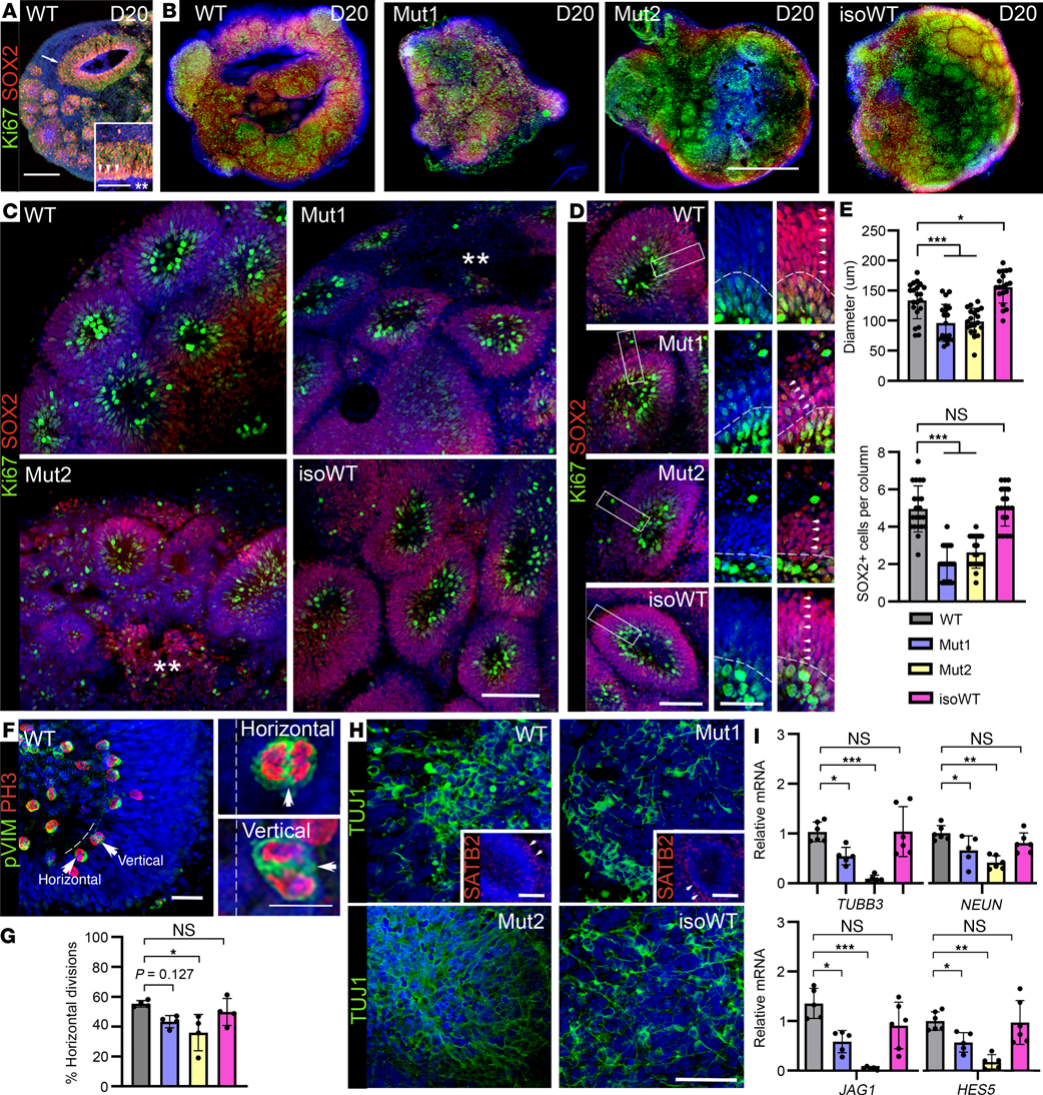
MCT8-deficient COs exhibit impaired early neurodevelopment and reduced expression of neuronal markers. (**A**) Confocal images of cryostat 10 μm–thick slice of a WT D20 CO. The arrow points to dense vertical columns of SOX2/Ki67^+^ cells, including a fluid-filled luminal compartment with an apical layer of progenitors (double asterisk and arrowheads in the inset). Scale bar 75 μm; inset in **A**: scale bar 35 μm. (**B**) Whole-mount immunolabeling of D20 control and MCT8-deficient COs. Scale bar 500 μm. (**C**) Representative circular rosette-like substructures (nuclear staining DAPI in blue). ** indicates areas of undifferentiated tissue; scale bar 150 μm. (**D** and **E**) Quantitation of the diameter of the cortical rosettes and number of layers formed by SOX2^+^ cells. Scale bar 150 μm; inset in **D**: scale bar 50 μm. Note how both parameters are reduced in MCT8-deficient COs when compared with controls. Values are mean ± SD of 4 COs per line (5–6 rosettes per CO). (**F**) Staining for phospho-histone H3 (PH3; red) and phospho-Vimentin (green) to mark neural precursor cells in mitosis, which primarily divide at the apical surface. Arrows in **F** and in the insets mark apical surface horizontal (0–30 degrees) and vertical (60–90 degrees) divisions. Scale bar 10 μm; insets in **F**: scale bar 5 μm. (**G**) Quantitation of neural precursors’ division orientation from 4 COs per line. (**H**) Whole-mount immunolabeling of D20 COs, Tuj1 staining in green, nuclear staining DAPI (blue) showing evenly distributed postmitotic neurons. Scale bar 100 μm; and insets in **H**: scale bar 150 μm. (**I**) Quantitation of the mRNA levels of the indicated genes in D20 control and MCT8-deficient COs. Note how the expression of genes involved in neurogenesis and neuronal differentiation is reduced in MCT8-deficient when compared with control COs. *n* = 5–6 RNA samples, each of them consisting of 4 pooled COs from either WT or MCT8-deficient COs; 1-way ANOVA and Tukey test were used for multiple comparisons; *P < 0.05, **P < 0.01, ***P < 0.001.

Control and MCT8-deficient D20 COs were able to initiate a normal neurodevelopmental pattern with multiple large and continuous small cortical units composed of a neural rosette constituting neuroepithelium-like structures similar to a ventricle (30). These neural rosettes were arranged in a laminar fashion, containing SOX2^+^ neural progenitors and Ki67^+^ proliferating cells (Figure 2, A–C), resembling the ventricular zone of the *fetal* cortex at about gestational week 6.5 (31). However, while WT and IsoWT COs formed neural rosettes with mean ± SD diameters of 144.5 ± 28.5 μm containing mean ± SD 5.3 ± 1.1 layers of SOX2^+^Ki67^–^ cells, MCT8-deficient COs formed smaller rosettes of 97.2 ± 26.4 μm with only 2.5 ± 1.0 layers of SOX2^+^ cells (Figure 2, D and E). These alterations indicate a cell proliferation defect in the early stages of corticogenesis. To explore this hypothesis, we traced back the neural precursor cells, staining them for the mitotic marker phospho-histone H3 (PH3) and phospho-vimentin (PVIM) (32). In control and MCT8-deficient COs, the majority of neural precursor cells were dividing at the apical surface, a typical feature of the neuroepithelium with apicobasal polarity (Figure 2F). We then measured the plane of nuclear migration (mitotic spindle) occurring during the translocation of the nuclei for mitosis and observed that control COs exhibited mainly horizontal divisions (52.5% ± 5.6%), which were less abundant in MCT8-deficient COs (40.4% ± 9.0%, on average; Figure 2G). During human brain development, horizontal divisions contribute to normal neurogenesis by regulating cortical expansion (33, 34). Thus, the lower percentage of horizontal divisions in MCT8-deficient COs may explain the observed reductions in the diameter and thickness of the cortical units of the MCT8-deficient COs.

We next performed immunofluorescence labeling and gene expression studies of D20 COs to test for the presence of TUJ1^+^ postmitotic neurons and evaluate the expression of markers for neuronal differentiation and mature neurons. Control and MCT8-deficient COs were populated with TUJ1^+^ postmitotic neurons (Figure 2H). Furthermore, neurons exhibited a primitive separation with a late-born superficial layer featuring SATB2^+^ cells (inset in Figure 2H) (35). However, MCT8-deficient COs exhibited significantly reduced mRNA levels of the neuronal markers *TUBB3* and *NEUN* and of the genes involved in neuronal differentiation *JAG1* and *HES5* (Figure 2I).

### MCT8-deficient COs presented an altered expression of TH transporters.

**Figure 3:**
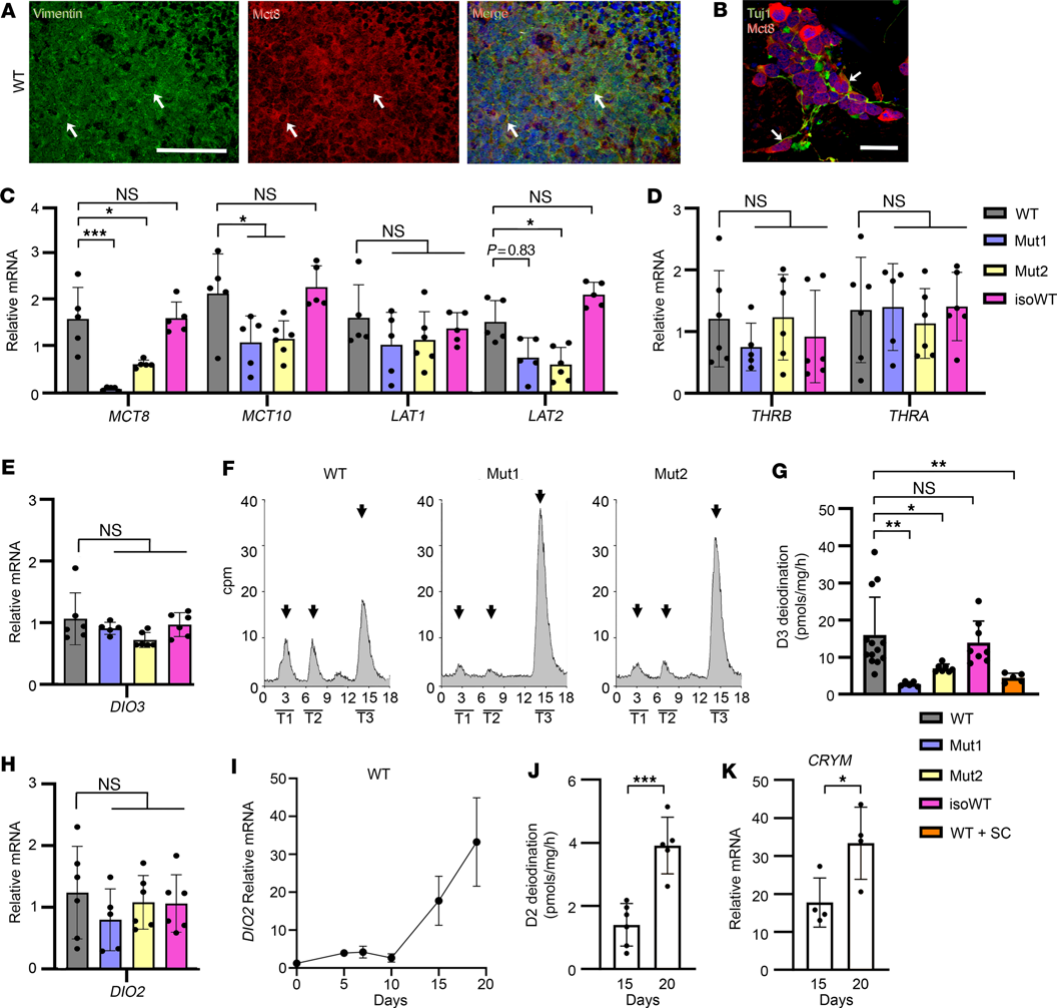
MCT8 mediates T3 transport in human neural cells. (**A** and **B**) Whole-mount immunolabeling of D20 WT COs, vimentin staining in green, MCT8 in red, colocalization indicated as yellow, nuclear staining DAPI as blue (**A**). (**B**) TUJ1 staining in green, MCT8 in red, nuclear staining DAPI in blue. Scale bar in **A**: 100 μm, in **B**: 10 μm. MCT8 staining was present in the outer cell membrane of neuronal elements (arrows). (**C**) Quantitation of the mRNA levels of the indicated TH transporters in D20 control and MCT8-deficient COs. (**D**) Same as **C** but for the genes *THRA* and *THRB*. (**E**) Same as **C** but for the genes *DIO3* and *DIO2*. (**E**) Same as **C** but for the gene *DIO3*. (**F**) Representative chromatograms of the medium after WT, Mut1, and Mut2 COs were incubated with T4-I^125^ for 3 hours. (**G**) Quantitation of the D3 deiodination in the indicated conditions; *n* = 5–13 D3 assays. (**H**) Same as in **C** but for the gene *DIO2*. (**I**) Relative *DIO2* mRNA levels in WT COs during their first 20 days in culture. (**J**) T4-I^125^ deiodination in D15 and D20 CO sonicates. (**K**) Relative *CRYM* mRNA levels in WT COs at D15 and D20. Expression values are mean ± SD of *n* = 3–6 RNA samples, each of them consisting of 4 pooled COs from either WT or MCT8-deficient COs; WT+SC: WT incubated with T4-I^125^ in the presence of 2 μM of Silychristin (SC). Two-tailed Student’s test for comparing D2 deiodination and relative mRNA expression between D15 and D20, and 1-way ANOVA and Tukey test were used for multiple comparisons; *P < 0.05, **P < 0.01, ***P < 0.001.

We first wanted to verify that MCT8 was present in neurons residing in D20 WT COs. Immunostaining for MCT8 and vimentin (a marker for neuronal cytoskeleton) revealed that MCT8 was evenly expressed in COs’ neurons (Figure 3A), including TUJ1^+^ postmitotic neurons (Figure 3B). Next, we examined the mRNA levels of TH transporters. Compared with D20 controls, MCT8-deficient COs exhibited a reduced expression of *MCT8*, *MCT10*, and *LAT2* and a similar expression of *LAT1* (Figure 3C). In these COs, we also measured the expression of TH nuclear receptors and found that control and MCT8-deficient COs exhibited a similar expression of *THRA* and *THRB* (Figure 3D).

### MCT8-deficient COs exhibit reduced D3-mediated metabolism.

Within the brain, neurons express high levels of the TH-inactivating deiodinase-3 (D3) that metabolizes T3 to T2 (36, 37), and glial cells express the activating enzyme D2, which metabolizes the prohormone, T4, to T3 (38–40). Here, we first studied changes in the *DIO3* expression (encoding D3) and found no differences between groups (Figure 3E). Next, we measured D3 activity as a proxy to assess T3 transport into the neural cells. D20 COs were incubated for 24 hours with T3-I^125^, resulting in a prominent peak of T2-I^125^ in the medium that was smaller in the MCT8-deficient COs (Figure 3F). Control COs exhibited D3 activity of 18.4 ± 7.7 pmol/mg/h, while MCT8-deficient COs had approximately 30% of that in WT (5.3 ± 3.1 pmol/mg/h) (Figure 3G). To test whether the decrease in the D3 activity observed in the MCT8-deficient COs was mediated via a defective MCT8 transport, we next incubated the control COs with T3-I^125^ in the presence of 2 μM of the highly selective MCT8 inhibitor Silychristin (WT+SC) (41), and indeed the D3 activity was decreased to 4.4 ± 3.0 pmol/mg/h (Figure 3G), a level similar to that observed in MCT8-deficient COs. Then, we studied changes in *DIO2* expression (encoding D2) and found no differences between groups (Figure 3H). *DIO2* expression peaked in D20 WT COs (Figure 3I), but we could not detect T3-I^125^ production in the medium after incubating D20 COs for 24 hours with T4-I^125^. *DIO2* is expressed in neural precursor cells (42–44), which also express the high-affinity T3-binding cytoplasmic protein μ-crystallin (CRYM) (45). Thus, it is conceivable that most of the T3-I^125^ generated via D2 may remain in the cytosol, trapped by this T3-binding cytoplasmatic protein. To explore this possibility further, we measured D2 activity in COs’ sonicates, which allowed us to identify D2-mediated T4-I^125^ to T3-I^125^ conversion of about 1.4 ± 0.7 pmol/mg/h in D15 COs, increasing to 3.9 ± 0.9 pmol/mg/h in D20 COs. Interestingly, these changes in D2 activity were paralleled by an approximately 89% increase in the expression of *CRYM* (Figure 3K).

### DITPA and TRIAC can trigger TH signaling in MCT8-deficient COs.

**Figure 4:**
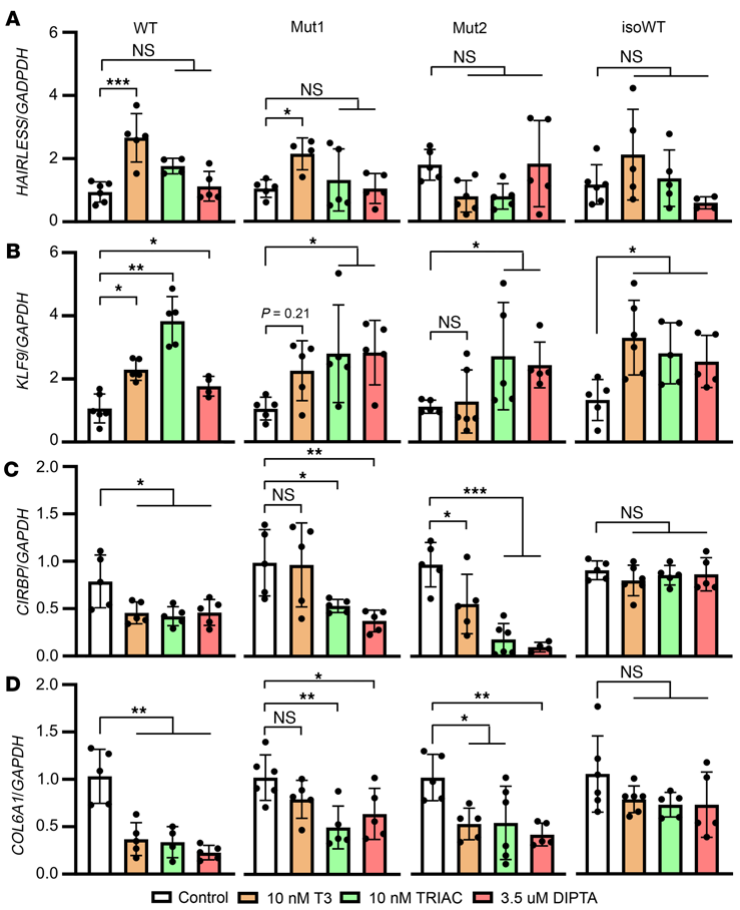
TRIAC and DITPA can trigger TH signaling in MCT8-deficient COs. (**A**–**D**) Changes in the mRNA levels of the indicated genes after 24 hours of the indicated treatments. The genes *HAIRLESS* and *KLF9* are upregulated by T3, while the genes *CIRBP* and *COL6A* are downregulated (99, 100). Values are mean ± SD of *n* = 5–6 RNA samples, each of them consisting of 4 pooled COs from either WT or MCT8-deficient COs; 1-way ANOVA and Tukey test were used for multiple comparisons; *P < 0.05, **P < 0.01, ***P < 0.001.

We next treated for 24 hours control and MCT8-deficient COs with 10 nM T3 and equivalent doses of DITPA (3.5 μM) or TRIAC (10 nM) and measured T3-responsive genes. The respective changes in mRNA levels in the COs are shown in Figure 4. For genes upregulated by T3 in control COs, *HAIRLESS* and *KLF9* (Figure 4, A and B), T3 increased (*P* < 0.05) the expression of *HAIRLESS* in Mut1 but did not affect Mut2 COs. Notably, T3 did not affect *KLF9* mRNA levels in MCT8-deficient COs, but treatment with both DITPA and TRIAC increased (*P* < 0.05) its expression in MCT8-deficient COs. For genes downregulated by T3 in control COs, *CIRBP* and *COL6A1* (Figure 4, C and D), T3 did not affect these 2 genes in Mut1 COs, but either DITPA or TRIAC reduced (*P* < 0.01) the *CIRBP* and *COL6A* mRNA levels in these COs. In Mut2, however, T3 downregulated the expression of these genes, and the response was more significant when Mut2 COs were treated with TRIAC or DITPA. Overall, these results indicate that the T3 signaling in the MCT8-deficient COs might be compromised and represent a proof of concept that the thyromimetic molecules TRIAC and DITPA can act in human neural cells despite a nonfunctional MCT8.

### Transcriptome analysis.

**Figure 5:**
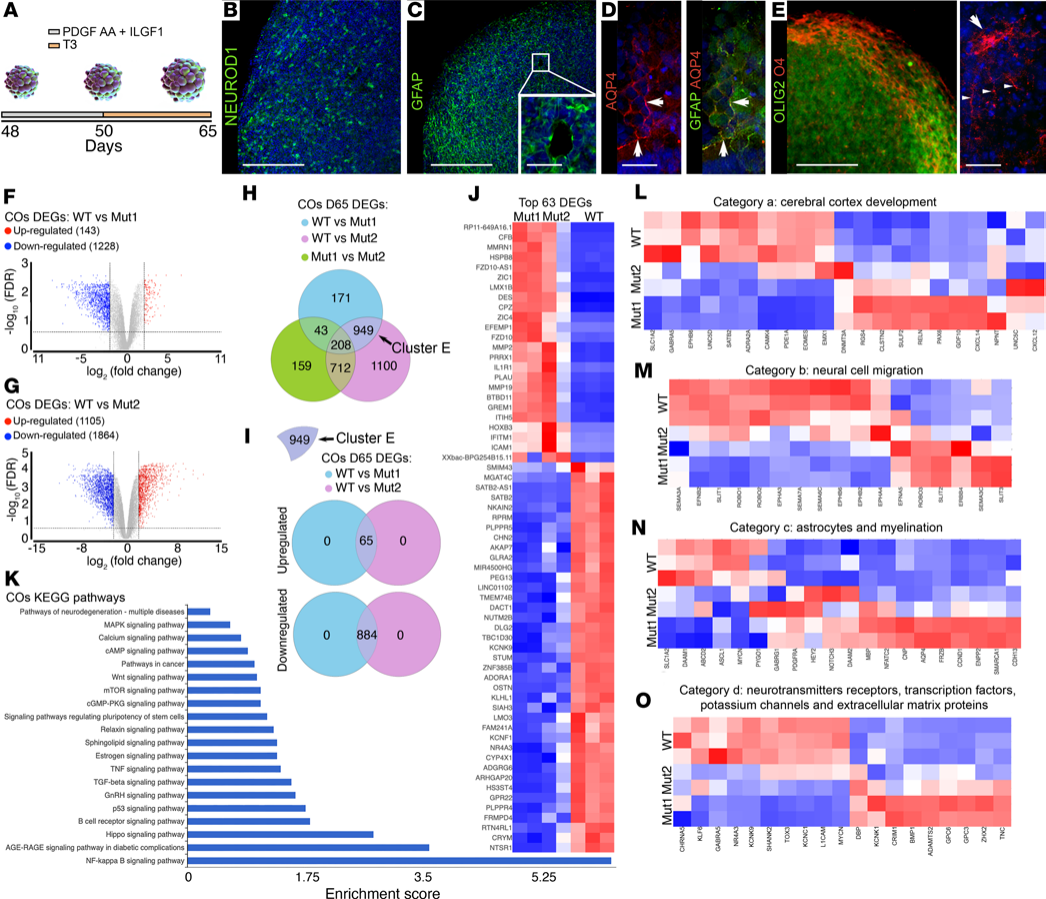
MCT8-deficient COs present an altered transcriptome. (**A**) Schematic of the protocol for COs’ maturation using platelet-derived growth factor AA and insulin-like growth factor 1. (**B**–**E**) Whole-mount immunolabeling of D65 WT COs containing differentiated NEUROD1^+^ neurons (**B**), GFAP^+^ neural precursor cells and GFAP-AQP4^+^ astrocytes (**C** and **D**), and OLIG2^+^ oligodendrocyte precursor cells and O4^+^ premyelinating oligodendrocytes (**E**); in blue is the nuclear staining with DAPI. **B**: scale bar 75 μm; **C**: scale bar 75 μm; inset in **C**: scale bar 25 μm; **D**: scale bar 40 μm; **E**: scale bar 75 μm; inset in **E**: scale bar 40 μm. Arrows in **D** indicate AQP4 staining in the membrane of GFAP^+^ cells. The arrow in the inset in **E** points to the cellular body of a representative oligodendrocyte; arrowheads point to its cellular processes. (**F** and **G**) Volcano plots showing the distribution of DEGs in WT vs. Mut1 (**F**) and in WT vs. Mut2 (**G**); each point represents the average of 3 (WT) or 2 (MCT8-deficient) samples consisting of 4 pooled COs for each transcript. (**H**) Venn comparison of WT vs. Mut1, WT vs. Mut2, and Mut1 vs. Mut2 DEGs. A common cluster (cluster E) between WT vs. Mut1 and WT vs. Mut2 containing 949 DEGs was identified. (**I**) Venn comparison of the 949 DEGs in cluster E shows similar transcriptomic changes in both MCT8-deficient COs. (**J**) Heatmap depicting the top 63 DEGs from cluster E. (**K**) KEGG pathway analysis of the top 63 DEGs in **J**. (**L**–**O**) Heatmaps of T3-regulated genes involved in cerebral cortex development (**L**), neural cell migration (**M**), astrocytes and myelination (**N**), and neurotransmitter receptors, transcription factors, potassium channels, and extracellular matrix protein (**O**). DEG thresholds (FDR ≤ 0.25 and fold-change ± 4) were identified by gene set–specific analysis in the Partek Flow platform.

To assess the effect of the T3 treatment (from D50 to D65) on the maturation of control and MCT8-deficient COs, we validated the presence of neurons, astrocytes, and oligodendrocytes in D65 COs by immunofluorescence imaging (Figure 5, B–E). D65 COs exhibited an evenly distributed population of NEUROD1^+^ differentiated neurons (Figure 5B), GFAP^+^ cells with 2 different morphologies resembling glial precursor cells (Figure 5C), and astrocytes in culture coexpressing aquaporin 4 (AQP4; a water channel critical for astrocyte function; Figure 5D). In addition, D65 COs contained abundant populations of OLIG2^+^ oligodendrocyte precursor cells and O4^+^ premyelinating oligodendrocytes (Figure 5E). In addition, we assessed the mRNA levels of *OLIG2* (markers of oligodendrocyte precursor cells) and *MBP* (markers of myelinating oligodendrocytes). Analysis of D50 versus D65 COs showed that the *OLIG2* mRNA levels increased similarly in WT (72.9% ± 25.7%) and Mut1 (68.5% ± 21.1%) and to a lesser extent in Mut2 COs (38.4% ± 12.9%). The levels of *MBP* mRNA increased at a similar rate (61.2% ± 10.8% on average) in both control and MCT8-deficient COs (Supplemental Figure 2, A and B).

The discovery that MCT8 deficiency impairs T3 uptake and action in COs (Figures 3 and 4), combined with the fact that T3 is required to promote the maturation of COs, suggests a role for MCT8 in CO maturation. To explore the role of MCT8 in COs’ maturation, we next performed RNA-Seq in D65 WT, Mut1, and Mut2 COs (after 15 days of treatment with 60 nM T3; these data sets clustered separately in a principal component plot; Supplemental Figure 3A).

Compared with controls, Mut1 and Mut2 COs were associated with the differential expression of 1,371 and 2,969 genes, respectively (1,228 and 1,864 downregulated and 143 and 1,105 upregulated; Figure 5, F and G, and Supplemental Tables 2 and 3). Among these genes, we identified a common cluster of 949 genes (cluster E; Figure 5H) that were either up- or downregulated (65 and 885, respectively, when comparing control versus Mut1 and versus Mut2; Figure 5I and Supplemental Tables 4 and 5). We then looked at the top 100 differentially expressed genes (DEGs) of cluster E and found that 63 were common for both MCT8-deficient COs (Figure 5J and Supplemental Table 6). Of these genes, the top gene *SATB2* is known to be regulated by T3 (46) and encodes a transcription factor that regulates neuronal differentiation and specification. *SATB2* is reduced/absent in some individuals manifesting developmental delay, intellectual disability, and severe speech delay (47) — common alterations in patients with AHDS. Another top gene, *KNCF1*, encodes a potassium ion channel involved in neurotransmitter release and neural excitation. Alterations in this gene have been associated with neurodevelopmental alterations in COs (48) and epilepsy (49). The gene *CRYM* (also studied in Figure 3) was also found to be among the top downregulated genes in MCT8-deficient COs. The remaining genes included *MMP2*, a modulator of neuronal precursor activity and cognitive and motor behaviors (50); *CFB*, a complement factor altered in neurologic diseases such as multiple sclerosis (51); and *HOXB3*, a critical choreographer of neural development (52). The biological interpretation of these changes was also studied using gene set enrichment analysis. We identified gene sets related to signaling pathways known to be regulated by T3 and relevant for cerebral cortex development, including MAPK, cAMP, Wnt, mTOR, TNF, TGF-beta, Hippo, and NF-kappa B (Figure 5K).

Next, we took advantage of our RNA-Seq data to assess T3-regulated genes (53, 54) proposed to have a role in cerebral cortex development (46) and noted significant expression changes between control and MCT8-deficient COs (Figure 5, L–O). We focused our attention on genes that are important for (a) normal development of the cerebral cortex, (b) neural cell migration, (c) astrocytes and myelination, and (d) neurotransmitter receptors, transcription factors, potassium channels, and extracellular matrix proteins (Figure 5, L–O).

With respect to category (a), the genes included *CAMK4* (abundant in cortical neurons and altered in hypothyroid animals) (55), *EOMES* (a regulator of cortical neurogenesis) (55), *GABRA5*, and *UNC5D* (enriched in immature embryonic subplate neurons) (46, 54); *ADRA2A*, *GDF10*, and *SULF2* (genes enriched in late fetal and postnatal neural cells); and *RGS4*, *EPHB6*, *CLSTN2*, and *DNMT3A* (genes expressed in immature Cajal-Retzius cells, ref. 56; alterations in *DNMT3A* cause progressive neurodegeneration, ref. 57). With respect to category (b), the genes included *EPHA3*, *EPHB6*, *ERBB4* (crucial for the migration, repulsion, and adhesion of neurons) (58); *SLIT1*, *SLIT2*, and *SLIT3*; and *ROBO1*, *ROBO2*, and *ROBO3*, as well as semaphorins like *SEMA3A*, *SEMA3C*, and *SEMA6C* (chemorepulsive molecules, some of them critical for axon pathfinding and neuronal maturation) (59, 60). With respect to category (c), the genes relevant for astrocytes included *SMARCA1* and *FRZB* (involved in the canonical and noncanonical WNT signaling pathways), *SLC1A2* (glutamate/aspartate transporter), *DAAM1* and *DAAM2* (modulating the WNT/JAK pathway), and *AQP4*. Some crucial DEGs for myelin were *MBP* (myelin constituent) and *CNP* (involvement of expansion of membranes during myelination). With respect to category (d), the genes included *GABRA5* (neurotransmitter γ-amino butyric acid receptor subunit) and *CHRNA5* (acetylcholine nicotinic receptor subunit) and the transcription factors *TOX3* and *KLF6* (for neuron survival) (61), *MYCN*, *EMX1*, and *ZHX2* (for progenitor cell division or maintenance) (62). The potassium channels, *KCNC1*, *KCNK9*, and *KCNK1* (influencing the biology of neurons and astrocytes) and the genes *ADAMTS2*, *GPC3*, *GPC6*, *BMP1*, *CRIM1*, *NAV2*, *L1CAM*, and *TNC* (extracellular matrix components important for an array of developmental processes including neuronal migration and axon outgrowth) (46).

In summary, among the genes studies in the 4 categories, 14 downregulated genes in MCT8-deficient COs are known to be transcriptionally upregulated by T3, and 15 upregulated genes in MCT8-deficient COs are known to be transcriptionally repressed by T3 (Supplemental Table 7). These findings strongly support our previous results showing that the T3 transport and action during the maturation of the MCT8-deficient COs was impaired, which led to an altered TH signaling in our MCT8-deficient COs.

## Discussion

The discovery that mutations in MCT8 are associated with AHDS revealed that transport across the blood-brain barrier and cell membranes is required for TH action in the brain (7, 63). However, critical questions remained unresolved. Here, we answer some of these questions and provide direct experimental evidence that reducing T3 transport into human neural cells leads to impaired T3 signaling and underlies the pathophysiology of the neurologic manifestations of AHDS. Our experiments with TH analogs demonstrated that DITPA and TRIAC could trigger improved responses (induction/repression of T3-responsive genes) in MCT8-deficient human neural cells, highlighting the promising potential for the early use of TH analogs in the treatment of patients with AHDS.

Having access to the human brain of MCT8-deficient patients would be the ultimate resource for the scientists who study the pathophysiology of this syndrome, but the invasive nature of the methods available limits such samples. Here, we overcame this hurdle by establishing an iPSC-derived model of COs that re-creates an MCT8-deficient environment mimicking the early fetal human brain development. To our knowledge, this is the first generation and characterization of MCT8-deficient COs derived from iPSCs obtained from patients with AHDS (7). Some of the critical functional features noted are i) EBs exhibited smooth and optically translucent edges, demonstrating neural induction; ii) COs developed neuroepithelial bud expansion and formed cerebral like structures of similar size; and iii) COs had multiple large and continuous small cortical units composed of a neural rosette arranged in a laminar fashion constituting neuroepithelium-like structures similar to a ventricle. In addition, control and MCT8-deficient COs exhibited similar mRNA levels of forebrain markers and proliferative and pluripotency markers (Figure 1). The presence of these features in MCT8-deficient COs indicates that a nonfunctional MCT8 does not compromise the potential of neural cells to initiate neurodevelopment.

However, as detailed herein, subsequent proliferation and differentiation of these precursors are altered in MCT8-deficient COs. For our studies, we utilized isogenic control COs (isoWT) and control COs obtained from iPSCs of the unaffected father of the patient with Mut2, with approximately 50% genetic similarity, in order to minimize the genetic background variance. Despite the difference in the genetic background between the 2 control lines, they showed similar outcomes in most of our experiments, indicating that the differences observed in MCT8-deficient COs are indeed due to the lack of functional MCT8. Our study is not without limitations as COs exhibit high heterogeneity, which may lead to low reproducibility. In our studies, this has led to variability in gene expression among replicates. Also, more physiological culture conditions are needed since the maturation of COs requires supraphysiological doses of T3 (60 nM) for long periods, which may partly overcome the lack of MCT8 by using alternative transporters.

Our findings support the hypothesis that an MCT8-mediated TH action is critical during early gestation, playing a role in human neurogenesis (43). This is evidenced by the smaller rosettes and thinner cortical units observed in MCT8-deficient COs. Alterations in these morpho-functional patterns have been associated with impaired proliferation of neural progenitor cells and even microcephaly in different cellular and animal models (64–68). In agreement, we observed that the plane of divisions of the neural precursor cells was different in control compared with MCT8-deficient COs, being predominantly horizontal in control COs. This finding is consistent with a study describing similar alterations in the neural progenitors during the early development of the optic tectum of chickens with a reduced expression of *MCT8* (18). The presence of these alterations in MCT8-deficient COs highlights the need for MCT8-dependent TH uptake in neural precursor cells and clarifies the importance of local TH action to establish the layered structure of the human forebrain during early embryonic development. That these alterations may affect neural differentiation is further evidenced by the consequential reduced expression of the neural differentiation markers *JAG1* and *HES5* and of the neuronal markers *TUBB3* and *NEUN* in MCT8-deficient COs and by a study analyzing brain and cerebellum sections from an MCT8-deficient fetus and identifying abnormalities in the density of neurons (8). Similar evidence was found in MCT8-knockin mice (P235L) that exhibit fewer neurons in the layers I–IV of the somatosensory cortex (69).

The present studies indicate that MCT8 is responsible for the bulk of the T3 transport across the membranes of the human neural cells residing in COs. This is illustrated by the reduced D3-mediated T3-I^125^ to T2-I^125^ conversion in MCT8-deficient COs. The peak of T2-I^125^ indicates the uptake of T3-I^125^ into the neural cells, its metabolism, and the release of T2-I^125^ to the medium. In agreement, studies in MCT8-deficient neural cells show an altered uptake and efflux of TH (13). The low level of D3 metabolism observed in MCT8-deficient COs may be attributed to some residual MCT8 function or to additional transporters (70, 71). The latter is less likely to be relevant, given (i) that SC blunted the D3 metabolism in the control line, resulting in low (but not fully suppressed) D3 activity; (ii) that Mut2 (missense mutation) exhibited more D3 activity than Mut1 (nonsense mutation; truncated protein); and (iii) that MCT8-deficient COs exhibited reduced MCT10 and *LAT2* and similar *LAT1* mRNA levels, suggesting an inability of the TH transport system to compensate for the absence of MCT8-dependent transport. This is consistent with observations in other MCT8-deficient, iPSC-derived brain microvascular endothelial like cells carrying a different mutation (72), although a decrease in mRNA levels of alternative transporters was not reported in iPSC-derived, MCT8-deficient neural cells (13). Conversely, in Mct8-KO mice, the expression of alternative TH transporters increases (73), likely contributing to the absence of an obvious neurological phenotype in these animals.

We have found that D2 is active in the early stages of COs’ maturation, when a high percentage of neural precursor cells expressing MCT8 is expected (Figure 3, A and B) (16, 74, 75). The D2-generated T3 seems to be of great value for these cells, as illustrated by the fact that we could not find D2-generated T3-I^125^ in the medium — as would be the case in mature astrocytes known to release the D2-generated T3 to the medium to act in neurons in a paracrine fashion (76, 77). Instead, we found that the elevated expression of the cytoplasmatic protein *CRYM* (reduced in MCT8-deficient COs; Figure 5J) likely functions to retain the D2-generated T3 in the cytoplasm of the neural precursor cells. The resulting buildup of T3 can trigger developmental programs in these cells. The relevance of this observation cannot be underestimated, as these neural precursor cells are the source of most human cortical neurons (78). Further research into the role of MCT8 and D2 in the biology of these cells is in progress.

These studies not only show that MCT8 is present in postmitotic neurons (Vimentin^+^ and TUJ1^+^ cells) residing in the COs but also show that T3 signaling is altered in MCT8-deficient COs, consistent with the observed reduction in TH transport. The lack of response to T3 of some of the genes studied in MCT8-deficient COs is significant. Conversely, in control COs, T3 resulted in the expected changes in up- and downregulated genes. Some of our results differ from a study on MCT8-dependent neural cells that showed similar dose-dependent T3-induced gene expression (13). A possible explanation for this discrepancy is that our T3 treatment lasted 24 hours versus an approximately 14-day chronic exposure to T3 in the latter. A limitation in both studies is the use of relatively high T3 concentrations (nM range), which do not recapitulate the lower in vivo conditions of free TH in cells (pM range) (79). The high doses of T3 may explain the unexpected increase in the mRNA levels of the gene *HAIRLESS* (and, to a lesser extent, the gene *KLF9*) in the Mut1 COs.

Regulation of gene expression is the foundation of our understanding of how TH acts in the developing brain. Most of what we know is inferred from studies in hypothyroid animals (80) and in mouse primary cultures (53, 54, 81) and by expressing a mutated version of the T3 receptor in specific mouse cells (82, 83). In our study, we have looked at approximately 65 T3-regulated genes that regulate critical processes of cerebral cortex development, and we found them to be altered in MCT8-deficient COs, making the case that TH signaling could be severely altered during the neurodevelopment of patients with MCT8-deficient syndrome. It is important to note that many of these genes are involved in critical functions, and the alterations of only a few of them can result in severe consequences; the control of *CAMK4* expression is a good example. *CAMK4* is known to be a transcriptional target of T3, and it is abundant in cortical neurons, having a crucial role in brain development and function (55, 84). Among *CAMK4* actions are the regulation of neuron differentiation and apoptosis and the regulation of dendritic growth and synaptic activity. We found that *CAMK4* was among the top downregulated genes in MCT8-deficient COs. Conversely, we also found some genes whose expression patterns were unexpected (i.e., induction/repression by T3 and the same responses in MCT8-deficient COs). This is the case of genes such as *MYCN* and *SLIT2*, which were upregulated by T3 in primary cultures and were also found to be upregulated in MCT8-deficient COs. These scenarios represent evidence that, in most cases, it is not easy to describe T3 actions linearly; in contrast, T3 action during brain development contributes to a network of interconnected pathways. Together, these findings in the transcriptome of control and MCT8-deficient COs suggest that in the maturation of the human brain, the changes in the transcriptome caused by T3 signaling are indeed significant and depend on MCT8.

The present studies also advance our understanding of how the TH analogs DITPA and TRIAC work in MCT8-deficient neural cells. The responses of the genes *KLF9*, *CIRBP*, and *COL6A1* to these TH analogs were significant (Figure 4, B–D). It was difficult to predict which TH analog would be more effective since DITPA and TRIAC have no preference for any TH nuclear receptors. We found that both TH analogs elicited similar responses in both MCT8-deficient COs. In addition, the present study demonstrates that DITPA and TRIAC can enter human neural cells via an MCT8-independent mechanism. This is illustrated by the fact that the Mut2 COs and isoWT responded similarly to the same dose of the analogs. The physiological implications of these findings are considerable, as they validate our species-specific model for the early development of the brain in an MCT8-deficient environment while showcasing the potential effectiveness of these TH analogs. Considering that (i) postnatal treatment with these analogs did not ameliorate the neurological symptoms and that (ii) when treating a mother carrying an MCT8-deficient fetus from 18 weeks of gestation until birth at 35 weeks (85), it improved the neuromotor and neurocognitive function, our study supports the idea that early prenatal treatment with TH analogs that can be available to fetus when given to the mother (86) may further rescue the phenotype.

In conclusion, here we provided definitive evidence that MCT8 is critical for early neurogenesis, mediating the bulk of the T3 uptake into developing human neural cells. Such perturbations and altered mechanisms likely play a key role in the pathogenesis of AHDS. Our studies validate DITPA and TRIAC as treatments that can elicit thyromimetic responses in human MCT8-deficient neural cells. Because of the interspecies differences in the expression of TH transporters in the brain, our studies using human MCT8-deficient COs carry significant physiological and clinical implications for how MCT8 loss-of-function mutations relate to the neurological manifestations seen in patients while providing the groundwork to develop new treatments.

## Methods

### Sex as a biological variable.

Our study exclusively examined cell lines derived from male patients and one from his father because the disease modeled is only relevant in males.

### Cell lines and maintenance.

iPSC lines were obtained from the Cedars-Sinai Regenerative Medicine Institute from MCT8-deficient individuals we have previously identified (7, 88). The 2 lines derived from fibroblasts of the patient with MCT8 deficiency were line CS58iMCT8 with MCT8 A404fs416X, here Mut1, and line CS01iMCT8 with MCT8 P321L, here Mut2. Mut1 is a nonsense mutation, causing a frameshift that results in a large deletion. Mut2 causes a missense mutation, resulting in a substitution of a proline for a leucine in position 321. Both mutations fully inactivate the transporter function (89, 90) and lead to similar clinical manifestations, including severe neuropsychomotor retardation and abnormal TH parameters. Additionally, we obtained 2 cell lines without *MCT8* gene mutations, namely CS01iMCT8cor, an isogenic line of Mut1 in which the mutation has been corrected by CRISPR/Cas9, here isoWT, and a line derived from the patient’s father, CS03iCTR, here WT, both serving as controls (Supplemental Figure 1A). The presence of the respective *MCT8* gene mutations and the correction by CRISPR/Cas9 in the specific iPSC lines have been verified by genomic DNA sequencing (Supplemental Figure 1B). The karyotype and pluripotency of each iPSC line were verified as previously described (13) and were regularly checked to rule out mycoplasma contamination (Supplemental Figure 1C). iPSCs were maintained on Matrigel-coated, 6-well plates in mTESR^+^ medium (unless otherwise indicated, all the culture medium and reagents were obtained from STEMCELL Technologies) at 37°C, 5% CO_2_, and were passaged every approximately 3.5 days (80% confluence) using RELESR solution.

### Generation of human brain organoids.

The methods to generate COs are highly reproducible (30, 91). On day 0, 80% confluent human iPSCs were dissociated using the Gentle Cell Dissociation Reagent, and approximately 9,000 cells/well were seeded in a 96-well, ultralow-attachment plate (Corning) in EB formation medium containing 10 μM Y-27632 (Tocris). On day 5, the medium was removed, and EBs were incubated with a neural induction medium. On day 7, EBs were kept on the 96-well plate, and the medium was supplemented with 2% Matrigel. On day 10, EBs were transferred into ultralow-attachment, 6-well plates kept in a maturation medium on an orbital shaker (75 rpm), with media changes every other day. From D20 onward, the maturation medium was switched to CO maturation medium containing BrainPhys and the supplements SM1, N2A, NEAA (Gibco), Glutamax (Gibco), insulin (MilliporeSigma), BME (MilliporeSigma), and 2 ng/mL brain-derived neurotrophic growth factor (Tocris). Every 3 days, half of the medium was replaced with a fresh one. For experiments with TH analogs, ~22-day-old COs were incubated in a maturation medium supplemented with 1% stripped serum (92) and with 10 nM L-T3, 10 nM TRIAC, or 3.5 μM DITPA (doses were adjusted based on their potency on cultured cells). Our D20 COs contained neural and glial precursor cells as well as dispersed neurons, establishing the structural and cellular framework for further maturation. To obtain COs populated by the 3 main cellular lineages of the brain, i.e., neurons, astrocytes, and oligodendrocytes, we followed established protocols (35, 93) and treated COs from day 48 to 50 with platelet-derived growth factor AA and insulin-like growth factor 1 (10 ng/mL; Tocris) and from days 50 to 65 with T3 (60 nM; MilliporeSigma) (Figure 5A). Treatment periods mirror the initial specification of oligodendrocyte precursor cells and mature oligodendrocytes (the myelin-generating cells) in the fetal human brain, which occurs at gestational weeks 10 and 14, respectively (35, 94).

### Quantitative real-time PCR.

Total RNA was extracted from 4 COs per group and treatment. mRNAs were treated with DNase (QIAGEN) and measured by quantitative real-time PCR as previously described (95). Briefly, total RNA was isolated using a QIAGEN RNeasy Mini Kit, according to the manufacturer’s instructions. The cDNA was prepared using a cDNA synthesis kit (Roche). Data were analyzed using the 2^−ΔΔCT^ method and displayed relative to an arbitrary value. The expression of the indicated genes was determined using specific primers (Supplemental Table 1). The expression of GAPDH was used as the internal control.

### Immunofluorescence and microscopy.

COs were fixed in 4% paraformaldehyde for 20 minutes, washed 3 times in PBS, and cryopreserved with a sucrose gradient (10→20→30%). Then, COs were embedded in OCT and cryosectioned at 20–30 μm using a sliding vibratome linked to a freezing unit. For whole COs’ immunofluorescence, after fixation, COs were kept in PBS on an orbital shaker overnight at room temperature. The next day, COs were incubated with 2% Triton X-100 in PBS (permeabilization solution) for 2 days while kept on the orbital shaker at room temperature. Immunofluorescence studies were performed as previously described (96), and COs were incubated with primary and secondary antibodies using the following dilutions: mouse monoclonal anti-Tuj1 1:1,000 (Bio-Techne; AF4320), anti-Sox2 1:1,000 (Abcam; 109186), anti-Ki67 1:1,000 (MilliporeSigma; MAB4190), anti-PVIM 1:400 (MBL; D076-3), anti-PH3 1:400 (Cell Signaling Technology; 9701S), anti-SATB2 1:400 (Abcam; ab92446), anti-Vimentin (Novus; NB300-223), anti-MCT8 antibody 1:400 (Atlas; HPA003353), anti-NEUROD1 1:400 (Bio-Techne; AF2746), anti-GFAP 1:200 (Novus; 05198), anti-AQP4 1:200 (Novus; 52872), anti-Olig2 (Abcam; ab109186), anti-O4 (Bio-Techne; MAB1326), Alexa Fluor 488–conjugated goat anti-mouse IgG 1:200 (Vector Laboratories; DK-2488), Alexa Fluor 594–conjugated horse anti-rabbit IgG 1:200 (Vector Laboratories; DK-1594), and Alexa Fluor 488–conjugated horse anti-rabbit IgG 1:200 (Vector Laboratories; DI-1788). Nuclei were counterstained with DAPI (1:10,000; Invitrogen; D1306). The images were analyzed with NIS-Elements AR (Nikon Instruments) or ImageJ software (NIH). Final figures were prepared with Adobe Photoshop (the illustration of COs shown in Figure 5A was generated with Adobe Photoshop AI).

### Iodothyronine chromatography using ultra-high-performance liquid chromatography.

COs were sonicated in 0.25 M sucrose in PE (0.1 M PBS, 1 mM EDTA) buffer and processed for deiodinase assays as previously described (97, 98). Before starting the deiodinase assays, T3-I^125^ and T4-I^125^ were purified on an LH-20 column. For measurements of D3 activity, ~20-day-old COs were incubated in 100 μL of medium containing 0.5 μM T3-I^125^. After 24 hours, the medium was sampled, mixed with 100 μL of 0.02 M ammonium acetate + 4% methanol + 4% PE buffer, and applied to the UPLC column (ACQUITY UPLC System, Waters). Fractions were automatically processed through a Flow Scintillation Analyzer Radiomatic 610TR (PerkinElmer) for radiometry. For measurement of D2 activity, T3-I^125^ production from 0.5 μM T4-I^125^ was determined in the presence of 10 nM T3 to saturate the D3. Deiodinase activities were normalized to the protein concentration (bicinchoninic acid method; Pierce, Thermo Fisher Scientific) and expressed as fractional conversion (pmol/mg/h).

### RNA-Seq and analysis.

Samples of total RNA were sent to the genomic facility at The University of Chicago for library preparation and sequencing. Libraries were paired-end sequenced with NovaSeq S4 (Illumina). Base calls and demultiplexing were performed with Illumina’s bcl2fastq software and a custom python demultiplexing program with a maximum of 1 mismatch in the indexing read. The FASTQ files were aligned to gencode hg38 transcriptome with STAR (v.2.7.8a) using the Partek Flow platform. Aligned reads were quantified to the annotation model (Partek E/M) and normalized to counts per million.

### Statistics.

All data were analyzed using Prism software (GraphPad). Unless otherwise indicated, data are presented as scatterplots depicting the mean ± SD. Comparisons were performed by a 2-tailed Student’s *t* test and multiple comparisons by 1-way ANOVA followed by Tukey test. A *P* < 0.05 was used to reject the null hypothesis. For RNA-Seq we identified DEGs with an FDR ≤ 0.25 and a fold-change ± 4.

### Study approval.

All experiments were approved by the Institutional Review Board at The University of Chicago (IRB 10255) and followed the American Thyroid Association Guide to investigate the TH economy and action in cell models (87).

### Data availability.

The Supporting Data Values file reports the data from Figures 1–4. RNA-Seq data presented in Figure 5 are available in the repository of Gene Expression Omnibus belonging to the National Center for Biotechnology Information (GSE253412).

## Author contributions

FSL conceptualized the study, conducted experiments, prepared figures, analyzed and interpreted the data, and prepared the manuscript. SE conducted experiments and prepared supplemental materials. SR, AD, and ACB interpreted the data and edited the manuscript. FSL and SR directed all the studies.

## Supplementary Material

Supplemental data

Unedited blot and gel images

Supplemental table 1

Supplemental table 2

Supplemental table 3

Supplemental table 4

Supplemental table 5

Supplemental table 6

Supplemental table 7

Supporting data values

## Version 1 02/20/2024

In-Press Preview

## Version 2 04/08/2024

Electronic publication
