# Supplementary material for: Impaired T3 uptake and action in MCT8-deficient cerebral organoids underlie Allan-Herndon-Dudley syndrome
Source: JCI Insight. 2024 Feb 20;9(7):e174645. doi: 10.1172/jci.insight.174645 (PMC11128209; doi:10.1172/jci.insight.174645)
Supplement: Supplemental data [file jciinsight-9-174645-s180.pdf]

Supplemental Figure 1

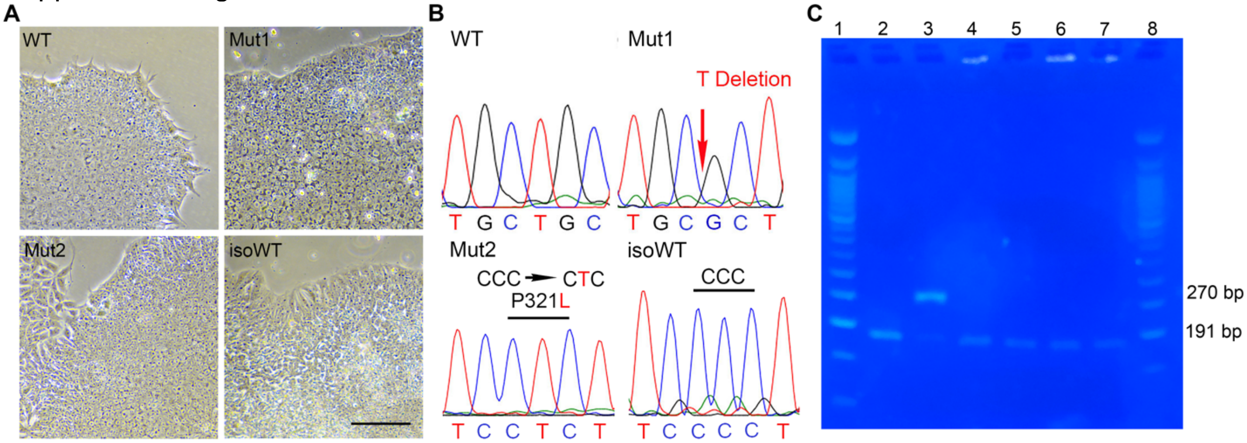

Supplemental Figure 1. Characterization of hiPSCs lines. A. Bright field images of the indicated hiPSCs colonies showed normal morphology, scale bars 100 µm. **B.** Sanger sequencing for exon 3 of the *MCT8* gene confirms the mutations in the Mut1 and Mut2 lines and the corrected mutation in isoWT. C. Agarose gel showing the results of a PCR-based mycoplasma detection test (Venor^{TM}GeM; Sigma Aldrich; MP0025) from the medium of the cultured hiPSCs lines and of the controls provided in the kit (positive was non-infectious DNA-fragments of *Mycoplasma orale*; negative was water). (1) 50 bp ladder, (2) negative control, (3) positive control, (4) Mut1 (5) Mut2 (6) WT (7) isoWT (8) 50 bp ladder. The presence of a 270 bp band indicates Mycoplasma contamination, while a 191 bp band indicates no contamination.

Supplemental Figure 2

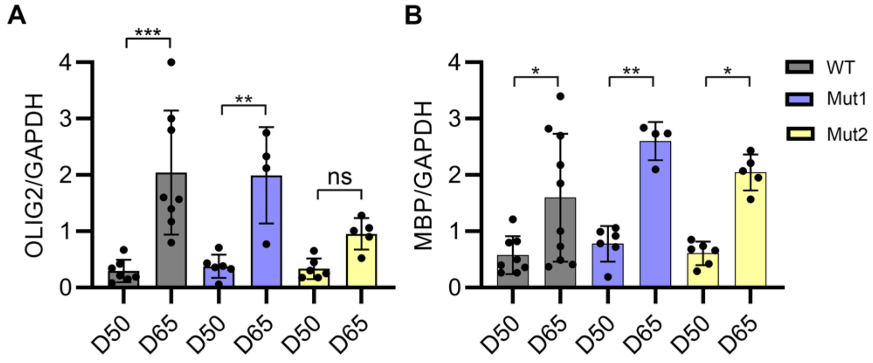

Supplemental Figure 2. Assessment of the maturation protocol. Changes in the mRNA levels of the indicated genes between D50 and D65 COs. values are mean ± SD of n = 4-6 RNA samples, each of them consisting of 4 pooled COs from either WT or MCT8-deficient COs ; **P* <0.05, ***P* < 0.01, ****P* < 0.001; ns: non-significant.

Supplemental Figure 3

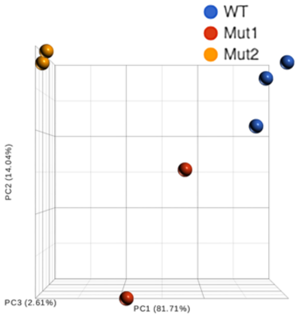

Supplemental Figure 3. Principal component plot illustrating differences between WT, MUT1 and MUT2 COs.
