# Supplementary figures and images for: Impaired T3 uptake and action in MCT8-deficient cerebral organoids underlie Allan-Herndon-Dudley syndrome
Source: JCI Insight. 2024 Feb 20;9(7):e174645. doi: 10.1172/jci.insight.174645 (PMC11128209; doi:10.1172/jci.insight.174645)

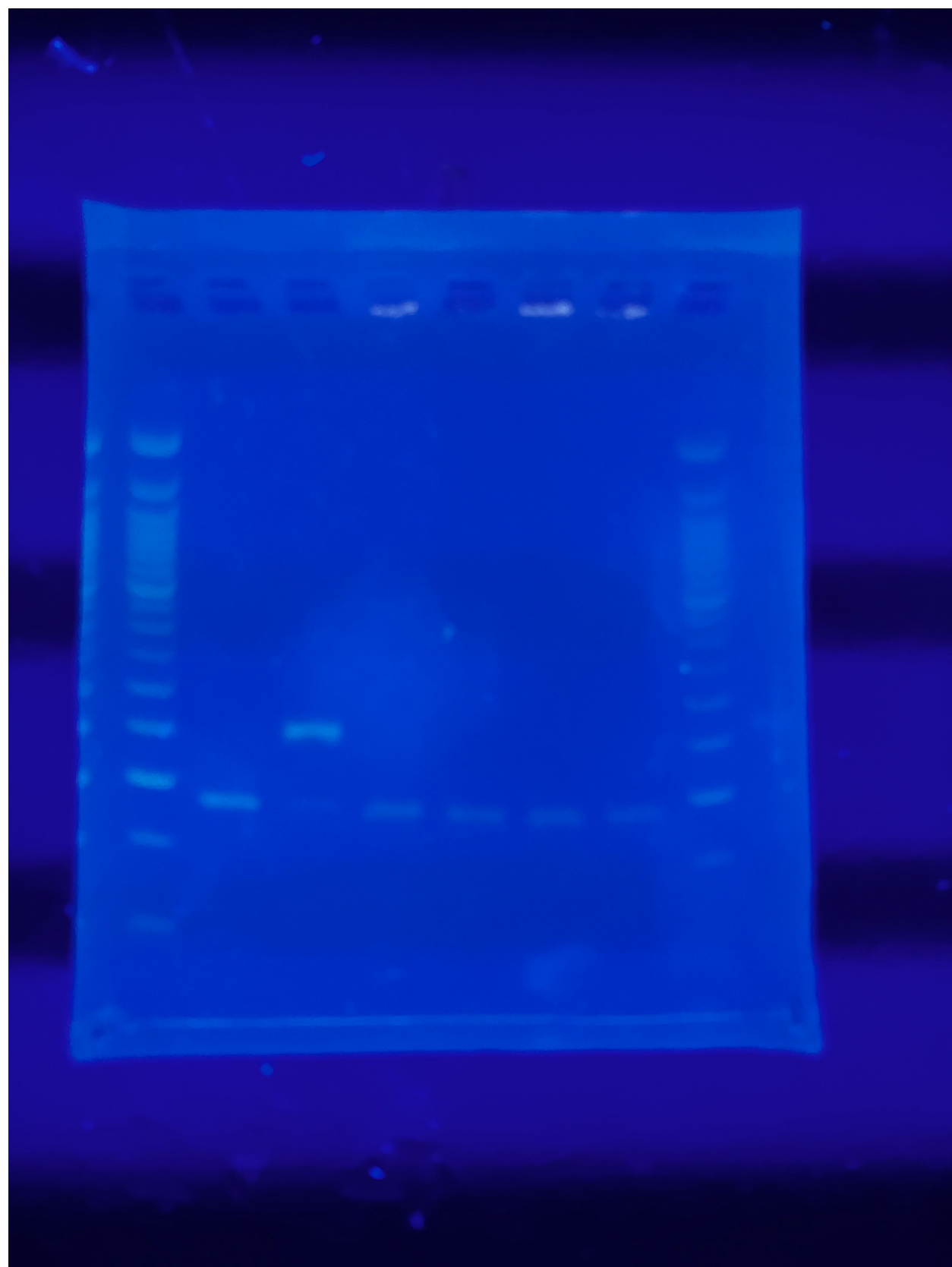

Supplement: Unedited blot and gel images [file jciinsight-9-174645-s181.pdf]
