# Supplementary material for: Impaired T3 uptake and action in MCT8-deficient cerebral organoids underlie Allan-Herndon-Dudley syndrome
Source: JCI Insight. 2024 Feb 20;9(7):e174645. doi: 10.1172/jci.insight.174645 (PMC11128209; doi:10.1172/jci.insight.174645)
Supplement: Supplemental table 1 [file jciinsight-9-174645-s182.docx]

| Supplementary Table 1. Primer Sequences | |  |
| --- | --- | --- |
| Gene | Forward Primer | Reverse Primer |
|  |  | |
| *FOXG1* | AACCTGTGTTGCGCAAATGC | AAACACGGGCATATGACCAC |
| *NKX2-1* | AGCACACGACTCCGTTCTC | GCCCACTTTCTTGTAGCTTTCC |
| *LMX1B* | AAACCCACGCAAACACACAC | TCTCTTTCTGACAAGGCAGGAC |
| *OCT4* | CTTCGCAAGCCCTCATTTCACCA | GCACTAGCCCCACTCCAACCTG |
| *SOX2* | TTCACATGTCCCAGCACTACCAGA | TCACATGTGTGAGAGGGGCAGTGTGC |
| *TUBB3* | ATCGGGGCCAAGTTCTGG | AAGAGATGTCCAAAGGCCCC |
| *NEUN* | CCAAGCGGCTACACGTCTC | CGTCCCATTCAGCTTCTCCC |
| *JAG1* | AGCCCTCATCCCTGTTACAA | TGCAAGGTCTCCCTGAAACT |
| *HES5* | GAGAAAAACCGACTGCGGAA | GAAGGCTTTGCTGTGCTTCA |
| *MCT8* | GCTTTCTGGCTCAGCTCAGG | TCCTCCACATACTTCATCAGGTGT |
| *MCT10* | GGCCGGATTGCAGATTATGT | CTATGGGAGCCATAATGGA |
| *LAT1* | CTCCTCACCCCCGTGCCGTC | GGCTTTCTGTGGCGCAGCC |
| *LAT2* | CCAGTGTGTTGGCCATGATC | GCAACCGTGACCCCATAGAA |
| *THRB* | AATCAGTGCCAGGAATGTCG | TCTTTTCTCCCGGTTCTCCTC |
| *THRA1* | GCTGCTAATGTCAACAGACCG | ATCATGCGGAGGTCAGTCAC |
| *DIO2* | GCTGCTGTTGAGCCGCTC | GCTCAGGGCTGGCAAAGTC3 |
| *DIO3* | GCTGGTTCTCAATTTCGGCA | GCTGTGGGATGATGTAGGGA |
| *HAIRLESS* | CCAAGTCTGGGCCAAGTTTG | TGTCCTTGGTCCGATTGGAA |
| *B-ACTIN* | GTGGACATCCGCAAAGACCT | ACTTGCGCTCAGGAGGAGC |
| *KLF9* | GGC TGT GGG AAA GTC TAT GG | AAG GGC CGT TCA CCT GTA TG |
| *CIRBP* | CGTCCATCTACAGACTTCCCA | TGGAGGGCTGAGTTTTGACA |
| *COL6A* | TCCAGCCCCTTCTTGATAGC | CAACCTGAGGGACAGGTACT |
| *PDGFRa* | TACACTTGCTATTACAACCACA | ATCCTCCACGATGACTAAAT |
| *OLIG2* | AAGGCAGTTGCTGTGGAAAC | GCAAACAGCTTAGCATTGCG |
| *SYT12* | CTGGGAGGTGGGTGTCTATG | CAGGCAGGCACTCTCTTCT |
| *GAPDH* | GGAGCGAGATCCCTCCAAAAT | GGCTGTTGTCATACTTCTCATGG |
