# Supplementary material for: Impaired T3 uptake and action in MCT8-deficient cerebral organoids underlie Allan-Herndon-Dudley syndrome
Source: JCI Insight. 2024 Feb 20;9(7):e174645. doi: 10.1172/jci.insight.174645 (PMC11128209; doi:10.1172/jci.insight.174645)
Supplement: Supplemental table 7 [file jciinsight-9-174645-s188.docx]

Supplemental Table 7. Summary of the changes in mRNA expression of the indicated genes in previous studies and in our MCT8-COs (compared to WT).

| Gene  *Observations | Effect of T3  (fold change) | Mut1 vs WT | Mut2 vs WT | Category | Reference | Primary culture |
| --- | --- | --- | --- | --- | --- | --- |
| *RELN** | $\uparrow$ (0.23) | $\uparrow$ | $\leftrightarrow$ | a | (1) | Cerebrocortical |
| *CAMK4* | $\uparrow$ (0.29) | $\downarrow$ | $\leftrightarrow$ | a | (1) | Cerebrocortical |
| *SEMA3C* | $\uparrow$(1.59) | $\uparrow$ | $\leftrightarrow$ | b | (1) | Cerebrocortical |
| *RGS4* | $\downarrow$(-0.98) | $\uparrow$ | $\uparrow$ | a | (1) | Cerebrocortical |
| *GDF10* | $\uparrow$(0.95) | $\uparrow$ | $\leftrightarrow$ | a | (1) | Cerebrocortical |
| *EFNB2* | $\downarrow$(-0.39) | $\downarrow$ | $\downarrow$ | b | (1) | Cerebrocortical |
| *EPHA4* | $\uparrow$(0.56) | $\downarrow$ | $\leftrightarrow$ | b | (1) | Cerebrocortical |
| *EPHB2* | $\uparrow$(0.42) | $\downarrow$ | $\downarrow$ | b | (1) | Cerebrocortical |
| *EPHA3* | $\uparrow$(0.37) | $\downarrow$ | $\downarrow$ | b | (1) | Cerebrocortical |
| *EPHB6* | $\uparrow$(0.32) | $\downarrow$ | $\downarrow$ | a / b | (1) | Cerebrocortical |
| *EFNA5* | $\downarrow$(-0.51) | $\uparrow$ | $\uparrow$ | b | (1) | Cerebrocortical |
| *SLIT2* | $\downarrow$(-0.39) | $\uparrow$ | $\uparrow$ | b | (1) | Cerebrocortical |
| *ROBO2* | $\uparrow$(0.28) | $\downarrow$ | $\downarrow$ | b | (1) | Cerebrocortical |
| *SLIT3* | $\downarrow$(-0.36) | $\uparrow$ | $\leftrightarrow$ | b | (1) | Cerebrocortical |
| *ROBO1* | $\downarrow$(-0.29) | $\downarrow$ | $\leftrightarrow$ | b | (1) | Cerebrocortical |
| *SEMA3A* | $\downarrow$(-0.18) | $\downarrow$ | $\downarrow$ | b | (1) | Cerebrocortical |
| *SEMA7A* | $\uparrow$(0.91) | $\downarrow$ | $\downarrow$ | b | (1) | Cerebrocortical |
| *SEMA6C* | $\uparrow$(0.79) | $\downarrow$ | $\leftrightarrow$ | b | (1) | Cerebrocortical |
| *SHANK2* | $\uparrow$(0.26) | $\downarrow$ | $\downarrow$ | d | (1) | Cerebrocortical |
| *GABRA5* | $\downarrow$(-0.32) | $\downarrow$ | $\downarrow$ | a / d | (1) | Cerebrocortical |
| *GABRG1* | $\downarrow$(-0.23) | $\leftrightarrow$ | $\leftrightarrow$ | c | (1) | Cerebrocortical |
| *CHRNA5* | $\uparrow$(0.38) | $\downarrow$ | $\downarrow$ | d | (1) | Cerebrocortical |
| *NOTCH3* | $\downarrow$(-0.52) | $\uparrow$ | $\leftrightarrow$ | c | (2) | Astrocytes |
| *CCND1* | $\downarrow$(-0.41) | $\uparrow$ | $\uparrow$ | c | (2) | Astrocytes |
| *PYGO1* | $\downarrow$(-0.25)  $\downarrow$(-0.39) | $\downarrow$ | $\leftrightarrow$ | c | (1)  (2) | Cerebrocortical Astrocytes |
| *FRZB* | $\uparrow$(0.8)  $\downarrow$(-1.32) | $\uparrow$ | $\leftrightarrow$ | c | (1)  (2) | Cerebrocortical  Astrocytes |
| *SARCA1* | $\downarrow$(-0.26)  $\downarrow$(-0.59) | $\uparrow$ | $\leftrightarrow$ | c | (1)  (2) | Cerebrocortical Astrocytes |
| *MYCN* | $\uparrow$(0.28)  $\downarrow$(-0.6) | $\downarrow$ | $\downarrow$ | c / d | (1)  (2) | Cerebrocortical Astrocytes |
| *NFATC2* | $\downarrow$(-0.54) | $\uparrow$ | $\uparrow$ | c | (2) | Astrocytes |
| *CDH13* | $\downarrow$(-0.34) | $\uparrow$ | $\leftrightarrow$ | c | (2) | Astrocytes |
| *DAAM1* | $\uparrow$(0.39) | $\downarrow$ | $\downarrow$ | c | (2) | Astrocytes |
| *DAAM2* | $\uparrow$(0.99)  $\uparrow$(0.92) | $\uparrow$ | $\uparrow$ | c | (1)  (2) | Cerebrocortical Astrocytes |
| *EMX1* | $\uparrow$(0.69) | $\downarrow$ | $\leftrightarrow$ | a | (1) | Cerebrocortical |
| *PAX6* | $\leftrightarrow$ | $\uparrow$ | $\leftrightarrow$ | a | (1) | Cerebrocortical |
| *SLC1A2* | $\uparrow$(0.43) | $\downarrow$ | $\downarrow$ | a / c | (1) | Cerebrocortical |
| *UNC5D* | $\downarrow$(-0.48) | $\downarrow$ | $\downarrow$ | a | (1) | Cerebrocortical |
| *SATB2* | $\downarrow$(-0.25) | $\downarrow$ | $\downarrow$ | a | (1) | Cerebrocortical |
| *PDE1A* | $\downarrow$(-0.34) | $\downarrow$ | $\leftrightarrow$ | a | (1) | Cerebrocortical |
| *SULF2* | $\uparrow$(0.23) | $\uparrow$ | $\uparrow$ | a | (1) | Cerebrocortical |
| *UNC5C* | $\downarrow$(-0.45) | $\leftrightarrow$ | $\leftrightarrow$ | a | (1) | Cerebrocortical |
| *ABCD2* | $\uparrow$(0.67) | $\downarrow$ | $\leftrightarrow$ | c | (1) | Cerebrocortical |
| *HEY2* | $\downarrow$(-0.94) | $\uparrow$ | $\uparrow$ | c | (2) | Astrocytes |
| *AQP4* | $\uparrow$(0.27) | $\uparrow$ | $\leftrightarrow$ | c | (1) | Cerebrocortical |
| *DNMT3A* | $\uparrow$(0.15) | $\uparrow$ | $\uparrow$ | a | (1) | Cerebrocortical |
| *CLSTN2* | $\uparrow$(0,23) | $\uparrow$ | $\uparrow$ | a | (1) | Cerebrocortical |
| *EOMES* | $\downarrow$(-0.67) | $\downarrow$ | $\downarrow$ | a | (2) | Astrocytes |
| *SLIT1* | $\downarrow$(-0.74) | $\downarrow$ | $\leftrightarrow$ | b | (2) | Astrocytes |
| *ASCL1* | $\downarrow$(-0.41) | $\downarrow$ | $\downarrow$ | c | (2) | Astrocytes |
| *NR4A3* | $\downarrow$(-0.33)  $\downarrow$(-0.67) | $\downarrow$ | $\downarrow$ | d | (1)  (2) | Cerebrocortical Astrocytes |
| *TOX3* | $\downarrow$(-0,25)  $\uparrow$(0.38) | $\downarrow$ | $\downarrow$ | d | (1)  (2) | Cerebrocortical Astrocytes |
| *KCNC1* | $\downarrow$(1.01) | $\downarrow$ | $\downarrow$ | d | (2) | Astrocytes |
| *DBP* | $\uparrow$ (0.86)  $\uparrow$ (0.79) | $\uparrow$ | $\uparrow$ | d | (2) | Astrocytes |
| *KCNK1* | $\uparrow$ (0.29)  $\uparrow$ (0.56) | $\uparrow$ | $\leftrightarrow$ | d | (1)  (2) | Cerebrocortical Astrocytes |
| *BMP1* | $\uparrow$(0.36) | $\uparrow$ | $\uparrow$ | d | (1) | Cerebrocortical |
| *CXCL14* | $\uparrow$(0.93) | $\uparrow$ | $\leftrightarrow$ | d | (1) | Cerebrocortical |
| *NPNT* | $\uparrow$(0.8) | $\leftrightarrow$ | $\leftrightarrow$ | a | (1) | Cerebrocortical |
| *CXCL12* | $\downarrow$(-0.66) | $\leftrightarrow$ | $\leftrightarrow$ | a | (1) | Cerebrocortical |
| *ENPP2** | $\uparrow$(0.41) | $\uparrow$ | $\uparrow$ | c | (1) | Cerebrocortical |
| *KLF6* | $\downarrow$(-0.31) | $\downarrow$ | $\downarrow$ | d | (1) | Cerebrocortical |
| *Kcnk9* | $\uparrow$(0.43) | $\downarrow$ | $\downarrow$ | d | (1) | Cerebrocortical |
| *ADAMTS2* | $\uparrow$(0.51) | $\uparrow$ | $\uparrow$ | d | (1) | Cerebrocortical |
| *GPC6* | $\uparrow$(0.33) | $\uparrow$ | $\uparrow$ | d | (1) | Cerebrocortical |
| *GPC3* | $\downarrow$(-0.86) | $\uparrow$ | $\uparrow$ | d | (1) | Cerebrocortical |
| *ZHX2* | $\uparrow$(0.28) | $\uparrow$ | $\leftrightarrow$ | d | (1) | Cerebrocortical |

Genes known to be downregulated by T3 that exhibit an increased (or unchanged) expression in MCT8-COs compared to WT are in red. Genes known to be upregulated by T3 that exhibited a decreased expression (or unchanged) in MCT8-COs compared to WT are in blue. Change in expression results from comparing mRNA levels with or without T3 treatment for 24h. ↔, no difference with T3 treatment; ↓, reduced expression with T3 treatment; ↑, elevated expression with T3 treatment. Data extracted from 1. Gil-Ibañez P, García-García F, Dopazo J, Bernal J, and Morte B. Global Transcriptome Analysis of Primary Cerebrocortical Cells: Identification of Genes Regulated by Triiodothyronine in Specific Cell Types. Cereb Cortex. 2017;27(1):706-17. 2. Morte B, Gil-Ibáñez P, and Bernal J. Regulation of Gene Expression by Thyroid Hormone in Primary Astrocytes: Factors Influencing the Genomic Response. Endocrinology. 2018;159(5):2083-92. In the studies done in cerebrocortical cells, the cultures exhibited 75% neurons and 15% astrocytes.
